# Radical polymerization approach for ring opened oxanorbornene anhydride based macromonomers

**DOI:** 10.1080/15685551.2017.1409475

**Published:** 2017-12-05

**Authors:** Ravichandran H. Kollarigowda, Pankaj Thakur

**Affiliations:** ^a^ Department of Chemical and Materials Engineering, University of Alberta, Edmonton, Canada; ^b^ Department of Chemistry, Shoolini University, Bajol, Solan, Himachal Pradesh, 173229, India

**Keywords:** Poly(oxanorbornene anhydride), controlled radical polymerization, comb shaped polymers, ring-opening metathesis polymerization

## Abstract

In this work, we synthesized end group functionalization of the cis-Norbornene-5-6-endo-dicarboxylic anhydride species via the ring-opening metathesis polymerization (ROMP) of oxanorbornene derivatives generated a chiseled poly(cis-Norbornene-5-6-endo-Dicarboxylic anhydride) acrylate macromonomer. Further, acrylate oxanorbornene based macromonomer further polymerized via reversible addition fragmentation chain transfer (RAFT) polymerization technique. Chain transfer is exhibited in the structure during the radical polymerizations so that free radical polymerization could also be used to comb structure copolymers with a PDI value below 1.2 with the help of acrylate oxanorbornene. Atomic force microscopy reveals the comb shape of branched polymer brushes structure. This method involves polymerizable end-group attachment to a macromonomer, and the backbone of the comb polymer is created in a second step of the polymerization. We believe that this kind of comb structured polymers can be considered for different biological applications.

## Introduction

The polymer either pendant to the polymeric chain or end group is as a result of the introduction of a particular form of functional moieties enable the controlled chain growth polymerization. This has contributed to the emergence of several techniques for intricate macromolecular architectures; for instance, hyperbranched, graft, star-shaped, or comb polymers [1–3]. Following grafting from and grafting onto, grafting through or the macromonomer method can be used for the synthesis of graft and comb polymers [1–5].

The typical approach of backbone synthesis is the reversible addition fragmentation chain transfer (RAFT); this method is referred to as RAFT and is used to polymerize macromonomers grounded on polyethylene oxide (PEO) [1–7]. A sterically engaging form of polymers, for example, poly(dimethyl siloxane [8], oligo(2-ethyl2-oxazoline [9], poly(benzyl-L-glutamate [9–11], or poly-(3-caprolactone) is applied to derive macro-monomers [11,12]. These polymers could either be co- or homopolymerized via RAFT polymerization.

**Scheme 1.**
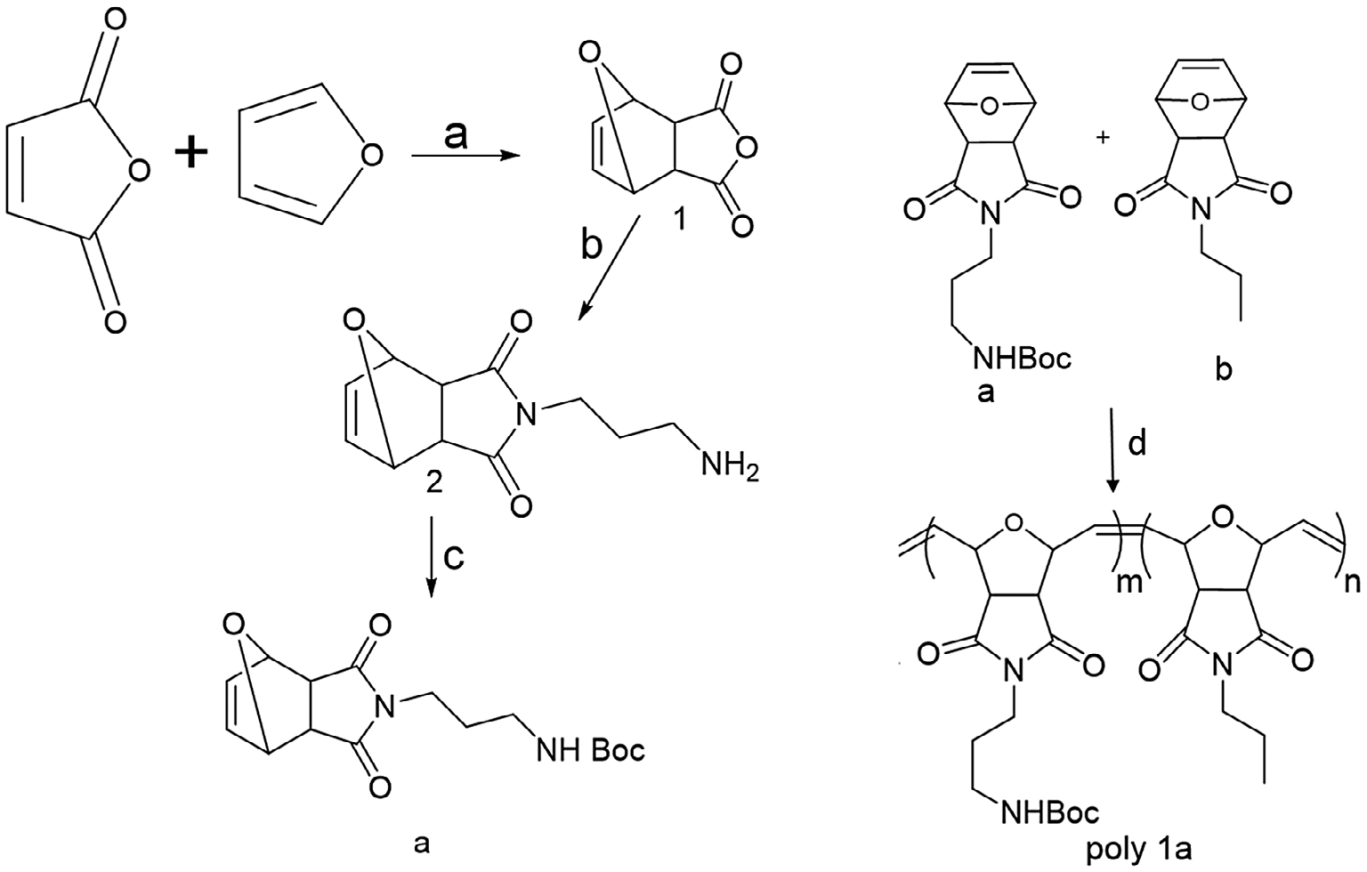
Synthesis of monomer a. Reaction scheme of diblock co-polymer synthesis (Poly 1a) via ROMP method using a ruthenium-based catalyst. Reaction conditions: (a) maleic anhydride (1.02 g, 10.4 mmol) and ethyl acetate (4.0 mL) cyclopentadiene (1 mL, 11.9 mmol) at RT for 48 hours. (b) 6.03 mmol of compound I, 2 eq wt of triethylamine, 1 eq wt 1,3 di-amino propane THF for 8 h. (c) 2 mmol of compound ii, 3 eq wt of TEA, 2 Eq wt of boc anhydride, THF at 70^°^C. (d) 9.43 mmol of compound a and 6.3 mmol of monomer B, 0.2 mml of ruthenium catalyst and THF for 5 h at RT.

Highly functional and well-defined block polymers are synthesized by a well-formulated ring-opening metathesis polymerization (ROMP) [13,14]. Monodispersed and stereoregular polymers are thermodynamically generated by the energetics of constrained bicyclic olefin monomer [15]. Norbornadiene, bicyclo[2.2.1] hepta-2,5-diene, and NBD are derivatives of ROMP of bicyclo[2.2.1]hept-2-ene (norbornene, NBE). They provide polynorbornadiene (PNBD) and polynorbornene (PNBE) (Scheme 1), and still encounter several challenges, which are experienced in spite of being researched for a while and having many publications. Chemical nature, concentration, the position of the substituent on the ring, temperature, and monomers are some of the physical [16], elements believed to help in the polymerization process. Grubb’s ruthenium-based elements show a diverse range of functionalization due to their dense resistance of heteroatom comprising groups, which interfered with previous catalysts [17–20].

In our work, we synthesized well-controlled block copolymer-based material on oxanorbornene anhydride derivatives and an oxanorbornene anhydride copolymer with ruthenium as a catalyst. Further, these ROMP polymers were acrylated by cleaving the boc and used for RAFT polymerization to form comb structured copolymers. Interesting to form comb polymer with the same side chains and distinct form of backbone, along with its effect on the macromolecules’ bottle brush in the solution is quite difficult. This might lead to differentiation of the polymer properties, for example, the way thermo-responsive materials behave in aqueous media, which has been the case for comb polymers with PEO side chains. From this viewpoint, the differentiation of the comb polymer backbone serves as a helpful element if the cloud point temperature (Tcp) of PE-tOx comb polymers is reduced in correlation to their linear analogs [21,22], this would be a result of PEtOx side chains of the improved “local concentration.” We could then evaluate if they are due the methacrylate backbone hydrophobicity.

**Scheme 2.**
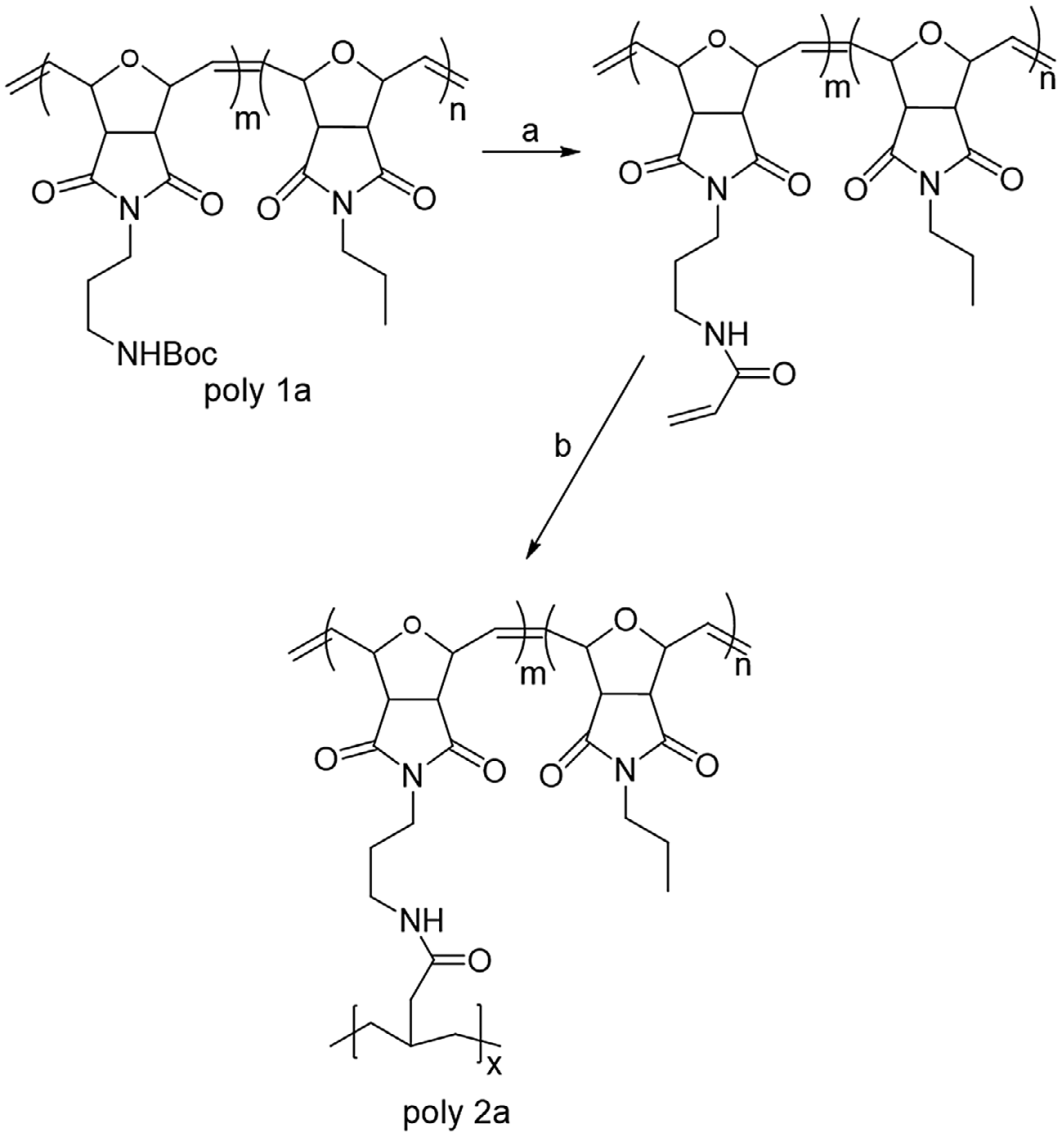
RAFT approach for the poly[(cis-Norbornene-5-6-endo-dicarboxylic anhydride) acrylate] comb gaft polymers. Reaction conditions: (a) [deprotection of boc- 6.3 mmol of poly 1a TFA (10 ml) and TIPS (3 ml) at RT for 24 hours]. 6.3 mmol of poly 1a (poly 1b and 1c), 0.049 mol of TEA, 0.055 mol acryloyl chloride at 0 ^°^C for 24 h. 6.3 mmol of poly 2a, 75.5 μmol CTA, 0.49 μmol AIBN and THF for 24 h at 70 ^°^C.

The approach used for the synthesis of the oxanorbornene anhydride side with an acrylate backbone and comb polymers include further end functionalization, which then leads to the synthesis of an acrylate end capped exo-7oxabicyclo [2.2.1] hept-5-ene-2,3-dicarboxylic anhydride macro-monomer after ROMP, followed by the second step. Block copolymerization of comb polymer synthesis is shown in Scheme 2 (Poly 2b), while kinetic and polymerization conditions are well-demonstrated in this research paper. Furthermore, atomic force microcopy (AFM) reveals the topography of the polymer brushes. Hence, we foreseen that these type biopolymers can be considered for different biological applications.

## Results and discussion

Diels-Alder reaction between maleic anhydride and furan prepared 3-dicarboxylic anhydride, and Exo-7-oxabicyclo[2.2.1]hept-5-ene-2.23 further, reacted with 1, 3 diaminopropane and boc anhydride was used to protect with the amine end group for further polymerization, as indicated in Scheme 1 (Supporting Information, Figure S1). Free amine does not allow polymerizing in the presence of a metal catalyst and can form a complex structure with ruthenium. Analysis of the 1H NMR affirmed product development of the monomer a (see Supporting Information, Figure S1).

By using ROMP, the diblock copolymer is synthesized based on the oxonorbornene anhydride. Diblock copolymer and homopolymer containing steric stabilizing blocks anchoring in 1:1 molar ratio had mild conditions, but were synthesized by ROMP as per the procedure in Scheme 1 and outlined in the experimental part SI. In an anaerobic glove box, the catalyst solution was prepared with water and air sensitivity of the Grubb’s catalyst. The Shlenk line was used to conduct polymerization reactions under an argon atmosphere [24]. The time difference between the add-on of the distinct monomers, the sequential order, and the well-defined block copolymers makes it difficult to prepare low PDI polymer [24].

**Figure 1.**
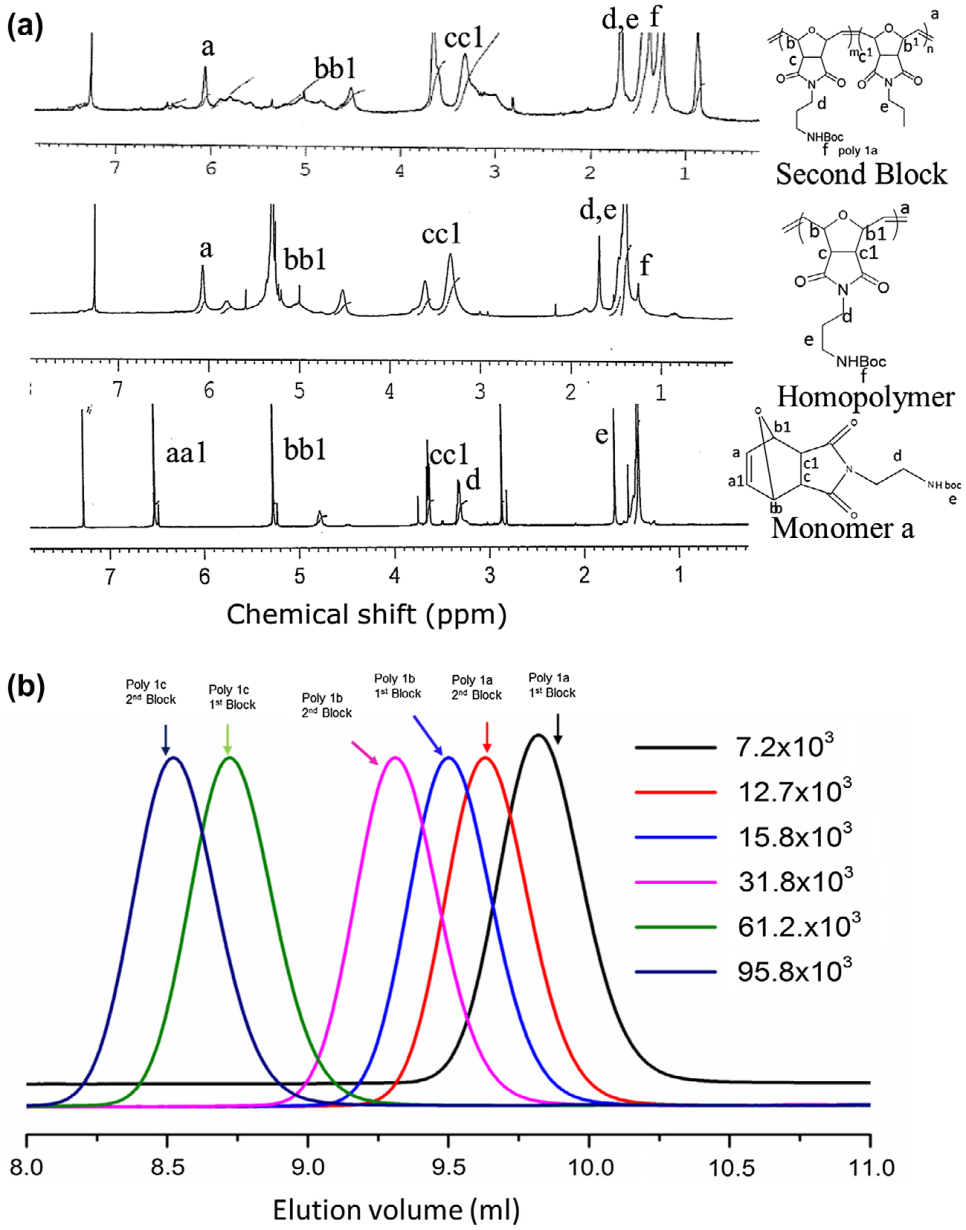
(a) 1H NMR spectra of monomer a. The homopolymer of oxanorbornene anhydride, poly (cis-Norbornene-5-6-endo-Dicarboxylic anhydride) and diblock copolymer of oxanorbornene anhydride derivative. (b) GPC chromatogram of blocks of poly 1a, b and poly 1c.

We used 1H NMR to determine the extent of polymerization of monomer a in CDCl3. This research study illustrates a peak at 5.12-5.24 ppm, which is a broad peak. It also shows a gradual vanishing of the vinyl monomer peaks at around 5.87–6.12 ppm (see Supporting Information, Figure S2a). It took about 5 h for completion of the homopolymerization; that is, for the complete monomer consumption. See Figure 1(a) (see supporting Information, Figure S2).

**Figure 2.**
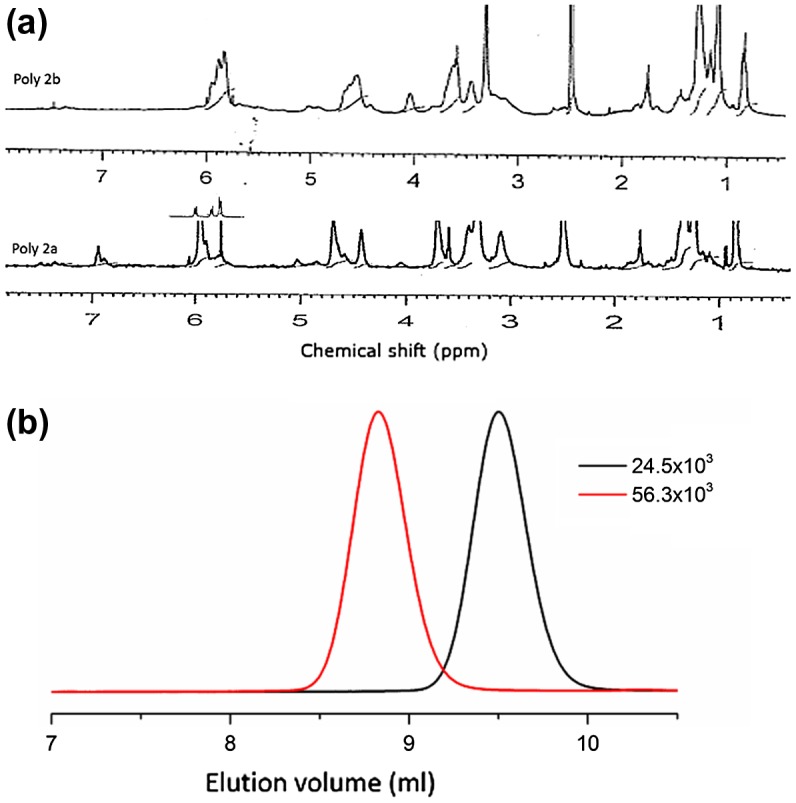
(a) RAFT polymerized of 1H NMR spectra of poly 2b and  poly 2a acrylate. (b) GPC chromatogram of synthesized polymer of poly 2b and 2c acrylate using poly 1a and Poly 1b.

**Figure 3.**
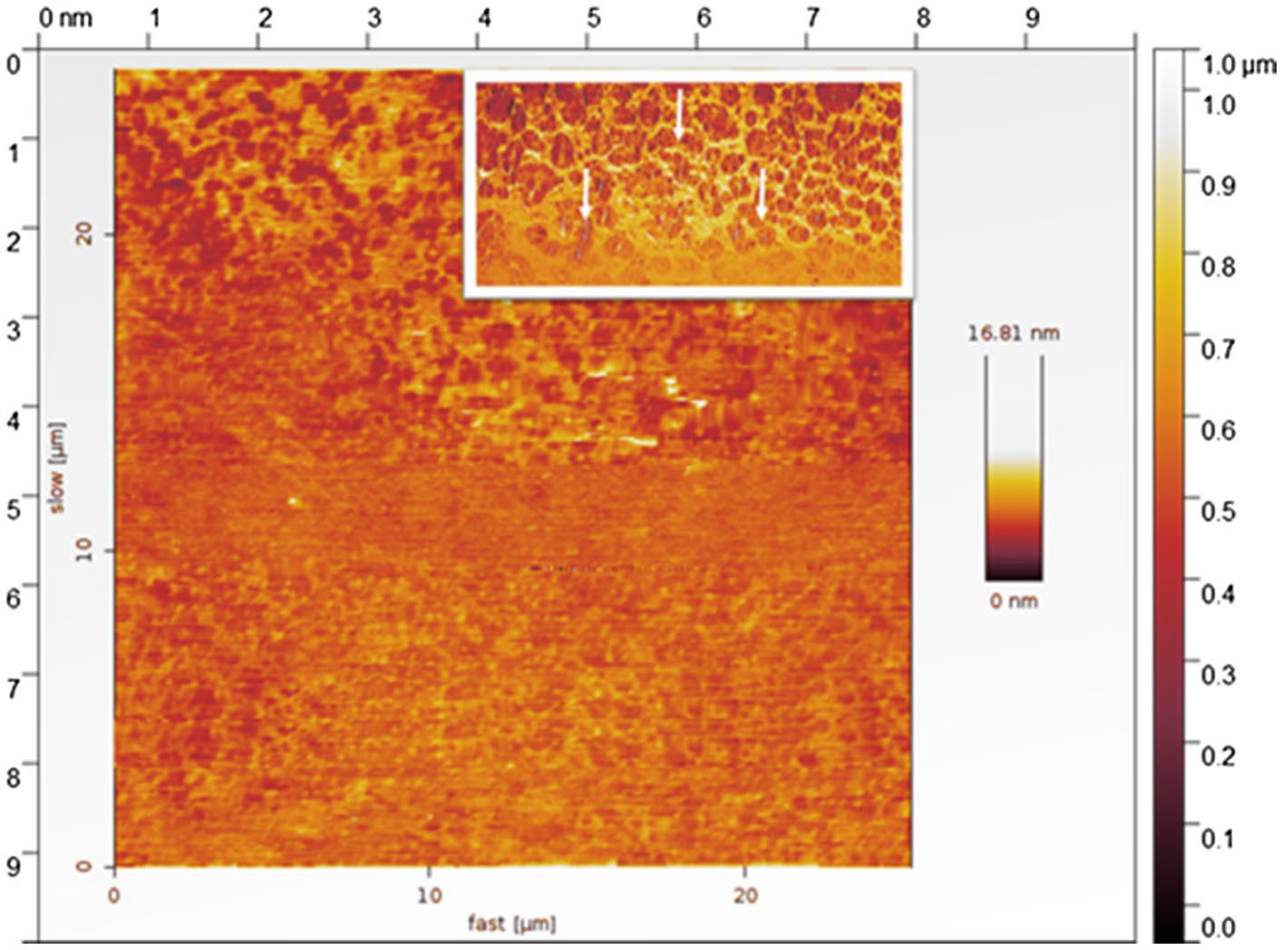
AFM image of RAFT grafted with Poly 2 (acrylated dimethyl-7-oxabicyclo [2.2.1] hept-5-ene exo 2,3dicarboxylate block copolymer. Inserted image 5 μm scale.

The propagation rate of the polymers relies on the polarity and stereochemistry exo/endo) of the replaced ligand because polymerization from the exo place of the norbornene vinylic bond is induced by the catalyst. Unsubstituted norbornene polymerization was found to be more reactive compared to the norbornene replaced at the 2-position [24,25]. In this case, the first block was substituted by the second monomer, and instead of the second block, oxanorbornene was added to synthesize the diblock copolymer. The molecular weight of diblock was achieved up to 95,000 g/mol with controlled polydispersity (see Figure 1(a and b)).

For our interest to see the fate of this polymers, we further cleaved the boc from block polymer of Poly 1a, Poly 1b and Poly 1c. Utilizing the free amine we introduced the acrylate group by simple acrylation method and used for further radical polymerization.

Interestingly, we noticed that further polymerization of Poly 2a by using AIBN as an initiator and radical polymerization occurred at 70 °C. Although Poly 1a has high molar mass, this polymer can further polymerize by using reactivity of the acrylate group. After this observation, we introduced RAFT approach to control the molecular weight by using RAFT agent to narrow down polymer dispersity index, See Scheme 2.

The monomer/initiator controlled the molecular weight of the polymers ([M]/ [I]) feed ratio. Monomer concentrations containing 0.15 M were kept in dry dichloromethane and polymerizations were performed at 30 °C. After all, first blocks were consumed, block copolymer synthesis was initiated and then the second monomer was added to the system. Longer response was needed for the block copolymer to generate high outcome with narrow polydispersity than the homopolymer [26].

In the end, by adding excess of ethyl vinyl ether quenched the polymerization by with constant stirring and then reaction mixture was precipitated in cold methanol. The system was redispersed in dichloromethane and reprecipitated in methanol. The polydispersity of the block copolymer was well controlled so that an increase in the second block lead to an increase in the polydispersity index, see Table 1.

**Table 1.** SEC (size exclusion chromatography) traces (THF) from the polymerization of GPC chromatogram of diblock polymers of Poly 1 kinetic studies.

Name	Mn × 10^3^	Mw × 10^3^	PDI	Conversion	Rutheniu
	(g/mol)^a^	(g/mol)^a^		(%)^b^	m (%)^c^
Poly 1a	1b-7.27	8.21	1.13	97	0.04
	2b-12.76	14.80	1.16	92	0.016
Poly 1b	1b-15.88	18.73	1.18	96	0.07
	2b-31.81	38.50	1.21	90	0.13
Poly 1c	1b-61.20	72.18	1.19	93	0.15
	2b-95.81	132.28	1.38	88	0.20
Poly 2b	28.47	34.45	1.21	68	–
Poly 2c	56.36	77.63	1.37	56	–

Notes:

THF RI detection, PS calibration was obtained from SEC. Ruthenium was calculated by ICP analysis, (1b-first block copolymer and 2b-second block copolymer).

a- Calculated by GPC

b- Calculated by using 1HNMR

c- Calculated by using ICP analysis

This polymer was further used for radical polymerization by modifying the pendant group of the primary polymer. Deprotecting the boc group of the main polymer chain and further  acrylated for the RAFT polymerization (synthesized poly 2b and 2c acrylate using poly 1a and Poly 1b).

Kinetic studies were performed during the RAFT polymerization of poly 1a in THF at 70 ^°^C with an M/TCA and applying trithiocarbonate and AIBN as the CTA and radical source respectively, because these conditions showed to be appropriate for the polymerization. From size exclusion chromatography (SEC) traces, conversions were determined to apply formed comb shaped polymer and peak areas of the macromonomer, after ensuring the accuracy of the employed approach by comparing the outcomes received by 1H NMR spectroscopy, see Figures 2(a and b) (Table S1).

The kinetic plot (Figure S3) is linear; various studies have indicated that polymerization rate decrease at high conversions hinting either towards the formation of persistent radicals or termination reactions that do not require to add further monomer [27,28]. In RAFT kinetics, the induction period is commonly observed at the start of the polymerization, although the primary purpose is not understood [29,30]. However, throughout the kinetic study of polymerization, unusual evolution of the 69 k Daltons molar mass occurred.

Although the comb polymer PDI remains lower than 1.3, the polymerization continues. However, the higher molecular weight of ring opened poly 1c did not polymerize. Here, we believe that the higher molar mass propagates the radical and forms complexes with residual of the ruthenium catalyst. To confirm the polymer topographical structure, we performed AFM, the polymer suspended solution was casted on glass surface and characterized by AFM by tapping mode. AFM revels that the polymer was crosslinked and appears as comb structure, see Figure 3.

Block copolymers of poly 1a, poly 1b and poly 1c did not show linear structure (data not shown), but after RAFT technique AFM confirms the comb shape structure and branched structure of polymers.

## Conclusions

In summary, we designed and synthesized diblock copolymer of oxanorbornene anhydride based derivative with low polydispersity index. Further poly (oxanorbornene) derivative was acrylated by deprotecting the boc from the amine group. An unusual radical polymerization character of well-defined oxanorbornene acrylate macromonomer was observed, while focusing toward comb polymers synthesis with an oxanorbornene anhydride side chains and acrylate backbone. Continuing monomer consumption was shown by kinetic studies of RAFT polymerization (synthesized poly 2b and 2c acrylate using poly 1a and Poly 1b), controlled molar mass with low PDI. Our study is the first effort to polymerize this polymer via radical polymerization. In addition to that atomic force microscopy reveals the comb shape structure of branched polymer brushes structure. We believe this opens up new platform to create a comb shape polymer brush in the polymer science field for different biological applications.

## Experimental

*Materials.* All raw materials and solvents were purchased from Sigma-Aldrich.

*Synthesis of monomer i and ii.* Compound i was prepared based on procedure in the literature [23,31,32]. The monoester of malonic acid was found from a previously reported procedure [33].

1 g (6.03 mmol) of compound 1 was dissolved in 25 ml of THF, while 2 equivalent weight mole of triethylamine was added into the solution. Moreover, 1 equivalent weight mole of 1,3 di-amino propane was dissolved and added in 25 ml of THF and added drop wise into the flask at 0 ^°^C. The reaction condition was maintained for throughout 8 h to avoid both side coupling with amine. The reaction mixture was washed with 1 N HCl saturated brine and water solution to remove impurities. The solvent was evaporated and obtained product was used for next step for Boc protection. 3 mol equivalent weight of triethylamine was added to the above mixture with corresponds to the weight of compound 2. 2 mol equivalent weight of Boc anhydride with respect to compound 2 was dissolved in 30 ml of THF and added drop wise to the reaction flask at room temperature [4]. Reaction was heated to 70 ^°^C and continued for 24 h. The reaction mixture was extracted with dichloromethane and washed with saturated solution of sodium bicarbonate, brine and water solution. Purification of the residue was done by column chromatography (SiO2, CH2Cl2/MeOH 100/1) gave 1.113 g (90%). 1H NMR: δ 1.25 (9H, s), 2.98–3.09 (2H, 3.04 (t, *J* = 10.1 Hz), 3.04 (t, *J* = 10.1 Hz)), 3.47–3.55 (2H, 3.51 (dd, *J* = 8.1, 5.3 Hz), 3.51 (dd, *J* = 8.1, 5.3 Hz)), 3.96–4.07 (2H, 4.02 (t, *J* = 10.1 Hz), 4.02 (t, *J* = 10.1 Hz)), 4.59–4.65 (2H, 4.61 (dd, *J* = 5.3, 1.8 Hz), 4.63 (dd, *J* = 5.3, 1.8 Hz)), 6.06–6.10 (2H, 6.08 (dd, *J* = 4.9, 1.8 Hz), 6.08 (dd, *J* = 4.9, 1.8 Hz)). m/z calcd for C16H22N2O5: 322.15: found: 322.105.

*Synthesis of monomer B.* 1 g (6.03 mmol) of compound 1 was dissolved in THF, while 2 equivalent weight mole of triethylamine was added into the solution. Moreover, 1 equivalent weight mole of 1-amino propane was dissolved in 25 ml of THF added drop wise into the flask at 0 ^°^C. The reaction condition was maintained for 8 h to avoid both side coupling with an amine group. The reaction mixture was washed with 1 N HCl saturated brine and water solution to remove impurities. Further compoud was purified by column chromatography (SiO2, CH2Cl2/MeOH 100/1) gave 1.13 g (85%). 1H NMR: δ 0.88 (3H, t, *J* = 6.6 Hz), 1.25–1.43 (2H, 1.34 (tq, *J* = 7.0, 6.6 Hz), 1.34 (tq, *J* = 7.0, 6.6 Hz)), 1.55–1.71 (2H, 1.63 (quint, *J* = 7.0 Hz), 1.63 (quint, *J* = 7.0 Hz)), 3.47–3.55 (2H, 3.51 (dd, *J* = 8.1, 5.3 Hz), 3.51 (dd, *J* = 8.1, 5.3 Hz)), 3.79–3.87 (2H, 3.83 (t, *J* = 7.0 Hz), 3.83 (t, *J* = 7.0 Hz)), 4.59–4.65 (2H, 4.61 (dd, *J* = 5.3, 1.8 Hz), 4.63 (dd, *J* = 5.3, 1.8 Hz)), 6.06–6.10 (2H, 6.08 (dd, *J* = 4.9, 1.8 Hz), 6.08 (dd, *J* = 4.9, 1.8 Hz)).

*Ring opening metathesis polymerization of dimethyl-7-oxabicyclo [2.2.1] hept-5-ene exo 2,3dicarboxylate.* Poly (dimethyl-7-oxabicyclo (2.2.1) hept-5-ene-2,3-dicarboxylic acid), 3, was prepared by dissolving 7-oxabicyclo [2.2.1] hept-5-ene-2,3- dicarboxylic acid, dimethyl ester, 2, (2 g, 9.43 mmol) in 30 ml THF. The ruthenium catalyst Ru(PPh3)2(Cl)2(CHPh) was added and the solution was stirred under nitrogen for 5 h [35]. The polymerization reaction was terminated by adding ethyl vinyl ether and the solution was stirred for 45 min. The polymer was precipitated by the addition of methanol to yield 90–95% of white polymer.

*Ring opening metathesis polymerization of dimethyl-7-oxabicyclo [2.2.1] hept-5-ene exo 2,3dicarboxylate-block- Monomer B.* Block copolymer was prepared by dissolving 7-oxabicyclo [2.2.1] hept-5-ene-2,3- dicarboxylic acid, dimethyl ester, 2, (2 g, 9.43 mmol) in 30 ml THF. The ruthenium catalyst Ru(PPh3)2(Cl)2(CHPh) was added and the solution was stirred under nitrogen for 5 h [35]. Next, degassed dry solution of monomer B 1.86 g (8.6 mmol) (second block) was added and the solution was stirred under nitrogen for 5 h. The polymerization reaction was terminating by adding ethyl vinyl ether and the solution was stirred for 45 min. The polymer was precipitated by the addition of methanol to yield 90–95% of white polymer.

*Deprotection of boc from the polymer and preparation of Poly 2a*. 10 g 6.3 mmol poly 1a was dissolved in 200 ml dichloromethane. TFA (10 ml) and TIPS (3 ml) were added to it. The reaction was continued overnight at RT. The reaction mixture was concentrated and the residue was dissolved in dichloromethane (100 ml). The solution was washed with brine (2 × 10 ml) and extracted with DCM. The residual solid was used for next reaction without further purification. Boc- deporotected polymer was dissolved in 200 ml of DCM. 5 ml (0.049 mol) of triethylamine was added into the above mixture. Then, 0.055 mol acryloyl chloride was dissolved in 50 ml dichloromethane and added dropwise for the reaction mixture at 0 ^°^C. The reaction was continued for 24 h at room temperature. At 24 h, the half of the solvent was evaporated and precipitated in cold ethanol. The polymer was filtered and washed with cold methanol to remove unreacted acryloyl chloride and other impurities, while the obtained polymer was dried with a vacuum at 50 °C. It was characterized by 1HNMR to confirm the acrylation of the double bond.

*Synthesis of Poly 2a (RAFT polymerization).* Poly 2 was dissolved in THF and AIBN as well as CTA were added from stock solutions to reach the feed ratios given in Table 1 and a monomer concentration of 1 mol L −1. The [CTA]:[AIBN] ratio was kept as 4:1 for all polymerizations [36]. 10 g (6.3 mmol) poly 2a was dissolved in 250 mL THF, 1.5 mL of a solution containing 30.5 mg (75.5 μmol)) CTA and 0.485 mL of a solution containing 7.6 mg (0.49 μmol) AIBN were added. After capping the vials and gently degassing the reaction mixtures by nitrogen bubbling, the vials were placed in an oil bath at 70 ^°^C for the desired reaction time. After cooling with tap water, the vials were opened and the samples were taken for SEC measurements to determine monomer conversion. Ethanol was removed under reduced pressure, the crude polymers were dissolved in THF and purified from residual Poly 2a by preparative SEC with THF as an eluent. The desired fractions were concentrated under reduced pressure, the OXN was precipitated into cold methanol and dried under reduced conditions.

## Supplemental data

Supplemental data for this article can be accessed https://doi.org/10.1080/15685551.2017.1409475

## Disclosure statement

There are no conflicts to declare.

## Supplementary Material

TDMP_1409475_Supplementary_Material.pdf

## References

[1] Han DH, Pan CY A novel strategy to synthesize double comb-shaped water soluble copolymer by RAFT polymerization. Macromol Chem Phys. 2006;207:836–843. 10.1002/(ISSN)1521-3935

[2] Khousakoun E, Gohy JF, Jérôme R Self-association of double-hydrophilic copolymers of acrylic acid and poly (ethylene oxide) macromonomer. Polymer. 2004;4:8303–8310. 10.1016/j.polymer.2004.10.020

[3] Laschewsky A, Mertoglu M, Kubowicz S, et al Lamellar structured nanoparticles formed by complexes of a cationic block copolymer and perfluorodecanoic acid. Macromolecules. 2006;39:9337–9345. 10.1021/ma061573 k

[4] Venkatesh R, Yajjou L, Koning CE, et al Novel brush copolymers via controlled radical polymerization. Macromol chem phys. 2004;205:2161–2168.

[5] Weber C, Babiuch K, Rogers S, et al Unexpected radical polymerization behavior of oligo (2-ethyl-2-oxazoline) macromonomers. Polymer Chem. 2012;3:2976–2985. 10.1039/c2py20479 g

[6] Krivorotova T, Vareikis A, Gromadzki D, et al Conventional free-radical and RAFT copolymerization of poly (ethylene oxide) containing macromonomers. Eur Polymer J. 2010;46:546–556. 10.1016/j.eurpolymj.2009.12.001

[7] Rinaldi D, Hamaide T, Graillat C, et al RAFT copolymerization of methacrylic acid and poly (ethylene glycol) methyl ether methacrylate in the presence of a hydrophobic chain transfer agent in organic solution and in water. J Polym Sci, Part A: Polym Chem. 2009;2009(47):3045–3055. 10.1002/pola.v47:12

[8] Hadjichristidis N, Iatrou H, Pitsikalis M, et al Macromolecular architectures by living and controlled/living polymerizations. Prog Polym Sci. 2006;31:1068–1132. 10.1016/j.progpolymsci.2006.07.002

[9] Weber C, Becer CR, Hoogenboom R, et al Lower critical solution temperature behavior of comb and graft shaped poly [oligo (2-ethyl-2-oxazoline) methacrylate] s. Macromolecules. 2009;42:2965–2971. 10.1021/ma8028437

[10] Runge MB, Dadsetan M, Baltrusaitis J, et al Development of electrically conductive oligo (polyethylene glycol) fumarate-polypyrrole hydrogels for nerve regeneration. Biomacromolecules. 2010;11:2845–2853. 10.1021/bm100526a 20942380 PMC3947846

[11] Kollarigowda RH, Mathews AS, Abraham S, et al A facile approach of light driven nanoassembly for the controlled accommodation of doxorubicin. Biomed Phys Eng Express. 2017;3(4):045006–45009. 10.1088/2057-1976/aa79b6

[12] Li J, Yi L, Lin H, et al Synthesis of poly (tert-butyl methacrylate)-graft-poly (dimethylsiloxane) graft copolymers via reversible addition-fragmentation chain transfer polymerization. J Polym Sci, Part A: Polym Chem. 2011;49:1483–1493. 10.1002/pola.24571

[13] Bielawski CW, Grubbs RH Highly efficient ring-opening metathesis polymerization (ROMP) using new ruthenium catalysts containing N-heterocyclic carbene ligands. Angew Chem. 2000;39:2903–2906. 10.1002/(ISSN)1521-3773 11028004

[14] Grubbs RH Olefin-metathesis catalysts for the preparation of molecules and materials (nobel lecture). Angew Chem Int Ed. 2006;45:3760–3765. 10.1002/(ISSN)1521-3773 16724297

[15] Rosebrugh LE, Ahmed TS, Marx VM, et al Probing stereoselectivity in ring-opening metathesis polymerization mediated by cyclometalated ruthenium-based catalysts: a combined experimental and computational study. J Am Chem Soc.2016;138:1394–1405. 26726835 10.1021/jacs.5b12277

[16] Kanai M, Mortell KH, Kiessling LL Varying the size of multivalent ligands: the dependence of concanavalin A binding on neoglycopolymer length. J Am Chem Soc. 1997;119:9931–9932. 10.1021/ja972089n

[17] Zhukhovitskiy AV, MacLeod MJ, Johnson JA Carbene ligands in surface chemistry: from stabilization of discrete elemental allotropes to modification of nanoscale and bulk substrates. Chem Rev. 2015;115:11503–11532. 10.1021/acs.chemrev.5b00220 26391930

[18] Iwasawa Y Inorganic oxide-attached metal catalysts. In Tailored Metal Catalysts, Springer Netherlands, 1986; 1–85.

[19] Elliott DC Catalytic hydrothermal gasification of biomass. Biofuels, Bioprod Biorefin. 2008;2:254–265. 10.1002/(ISSN)1932-1031

[20] Widegren JA, Bennett MA, Finke RG Is it homogeneous or heterogeneous catalysis? Identification of bulk ruthenium metal as the true catalyst in benzene hydrogenations starting with the monometallic precursor, Ru (II)(η6-C6Me6)(OAc) 2, plus kinetic characterization of the heterogeneous nucleation, then autocatalytic surface-growth mechanism of metal film formation. J Am Chem Soc. 2003;125:10301–10310. 10.1021/ja021436c 12926954

[21] Weber C, Becer CR, Hoogenboom R, et al Lower critical solution temperature behavior of comb and graft shaped poly [oligo (2-ethyl-2-oxazoline) methacrylate] s. Macromolecules. 2009;42:2965–2971. 10.1021/ma8028437

[22] Weber C, Remzi Becer C, Guenther W, et al Dual responsive methacrylic acid and oligo (2-ethyl-2-oxazoline) containing graft copolymers. Macromolecules. 2010;43:160–167. 10.1021/ma902014q

[23] Cooley JH, Williams RV Endo- and exo-Stereochemistry in the Diels-Alder Reaction: Kinetic versus Thermodynamic Control. J Chem Educ. 1997;74:582–585. 10.1021/ed074p582

[24] Jiao L, Bach T Palladium-catalyzed direct 2-alkylation of indoles by norbornene-mediated regioselective cascade C–H activation. J Am Chem Soc. 2011;133:12990–12993. 10.1021/ja2055066 21806073

[25] Sutthasupa S, Sanda F, Masuda T ROMP of norbornene monomers carrying nonprotected amino groups with ruthenium catalyst. Macromolecules. 2009;42:1519–1525. 10.1021/ma802243e

[26] Hadjichristidis N, Pitsikalis M, Iatrou H Synthesis of block copolymers. Block Copolymers. 2005:1–124.

[27] Alidedeoglu AH, York AW, McCormick CL, et al Aqueous RAFT polymerization of 2-aminoethyl methacrylate to produce well-defined, primary amine functional homo-and copolymers. J Polym Sci, Part A: Polym Chem. 2009;47:5405–5415. 10.1002/pola.v47:20

[28] Cortez-Lemus NA, Salgado-Rodríguez R, Licea-Claveríe A Preparation of α, ω-telechelic hexyl acrylate polymers with OH, COOH, and NH2 functional groups by RAFT. J Polym Sci, Part A: Polym Chem. 2010;48:3033–3051. 10.1002/pola.v48:14

[29] Bigot J, Charleux B, Cooke G, et al Synthesis and properties of tetrathiafulvalene end-functionalized polymers prepared via RAFT polymerization. Macromolecules. 2010;43:82–90. 10.1021/ma901809a

[30] hang X, Giani O, Monge S, et al. RAFT polymerization of N, N-diethylacrylamide: Influence of chain transfer agent and solvent on kinetics and induction period. Polymer. 2010;51:2947–2953.

[31] Barner-Kowollik C, Russell GT Chain-length-dependent termination in radical polymerization: Subtle revolution in tackling a long-standing challenge. Prog Polym Sci. 2009;34:1211–1259. 10.1016/j.progpolymsci.2009.07.002

[32] Barner-Kowollik C, Buback M, Charleux B, et al Mechanism and kinetics of dithiobenzoate-mediated RAFT polymerization. The current situation. J Polym Sci, Part A: Polym Chem. 2006;44:5809–5831. 10.1002/(ISSN)1099-0518

[33] Kollarigowda RH, Abraham S, Montemagno CD Antifouling Cellulose Hybrid Biomembrane for Effective Oil/Water Separation. ACS Appl Mater Interfaces. 2017;35:29812–29819. 10.1021/acsami.7b09087 28796485

[34] Constant C, Albert S, Zivic N et al Orthogonal functionalization of a fullerene building block through copper-catalyzed alkyne–azide and thiol–maleimide click reactions. Tetrahedron. 2014;70:3023–3029.

[35] Schrock RR Living ring-opening metathesis polymerization catalyzed by well-characterized transition-metal alkylidene complexes. Acc Chem Res. 1990;23:158–165. 10.1021/ar00173a007

[36] Chiefari J, Chong YK, Ercole F, et al Living free-radical polymerization by reversible addition− fragmentation chain transfer: the RAFT process. Macromolecules. 1998;31:5559–5562. 10.1021/ma9804951

