# Supplementary material for: Radical polymerization approach for ring opened oxanorbornene anhydride based macromonomers
Source: Des Monomers Polym. 2017 Dec 5;21(1):1–8. doi: 10.1080/15685551.2017.1409475 (PMC5812107; doi:10.1080/15685551.2017.1409475)
Supplement: TDMP_1409475_Supplementary_Material.pdf [file TDMP_A_1409475_SM7730.pdf]

Supporting Information

Radical Polymerization Approach for Ring Opened Norbornene Anhydride Based Macromonomers

Ravichandran H. Kollarigowda[a]*and Pankaj Thakur[b]

[a] *Department of Chemical and Materials Engineering, University of Alberta, 9211 - 116 Street, Edmonton, Alberta, CANADA, T6G 1H9.*

[b] *Department of Chemistry, Solan-Oachghat-Kumarhatti Highway, Bajol, Solan, Himachal Pradesh 173229, India.*

*kollarig@ualberta.ca, ravichandran.hk@gmail.com

Name	Page number
Synthesis of Monomer a	2
NMR of monomer a Figure S1	3
^{1}HNMR spectra monomer **a** and homopolymer spectra of Poly1a Figure S2	4
GPC calibration curve Figure S3.	5
Synthesis of Poly 2a (RFAT polymerization)	5

Experimental condition.

Materials. All the raw materials were purchased from Sigma-Aldrich. All solvents were purchased from Sigma-Aldrich and used as received.

Synthesis monomer i. Compounds was prepared according to the procedures reported in the literature.[1,2] The monoester of the malonic acid was obtained according to our previously reported procedure.[3]

Cyclopentadiene was previously prepared through the cracking of dicyclopentadiene and kept under cold conditions. In a 25 mL Erlenmeyer flask, maleic anhydride (1.02 g, 10.4 mmol) and ethyl acetate (4.0 mL) were combined, swirled, and slightly heated until completely dissolved. To the mixture, ligroin (4 mL) was added and mixed thoroughly until dissolved. Finally, cyclopentadiene (1 mL, 11.9 mmol) was added to the mixture and mixed extensively. The reaction was cooled to room temperature and placed into an ice bath until crystallized. The crystals were isolated through filtration in a Hirsch funnel. The product had the following properties: 0.47 g (27.6% yield) mp: 163-164 °C (lit: 164 °C). 1H NMR (CDCl3, 300 MHz) δ: 6.30 (dd, J=1.8 Hz, 2H), 3.57 (dd, J=7.0 Hz, 2H), 3.45 (m, 2H), 1.78 (dt, J=9.0,1.8 Hz, 1H), 1.59 (m, 1H) ppm. 13C NMR (CDCl3, 75Hz) δ: 171.3, 135.5, 52.7, 47.1, 46.1 ppm.

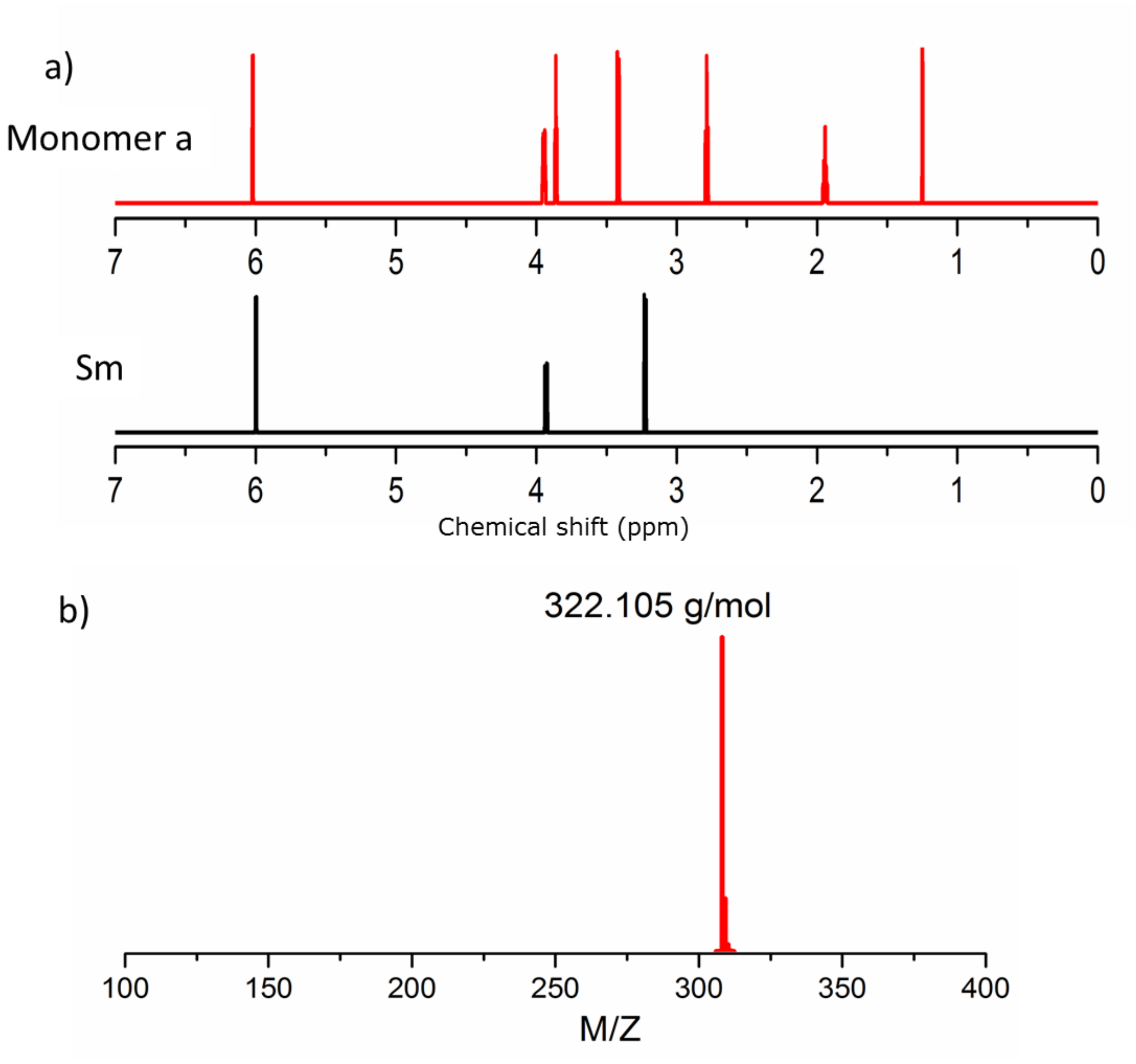

Figure S1. ^{1}HNMR spectra of synthesized monomer ii (a) and mass spectra of monomer a (b). NMR spectra were recorded on a Varian (300 MHz) NMR Spectrometer in DMSO-d6.

Ring opening metathesis polymerization of dimethyl-7-oxabicyclo [2.2.1] hept-5-ene exo 2,3dicarboxylate.

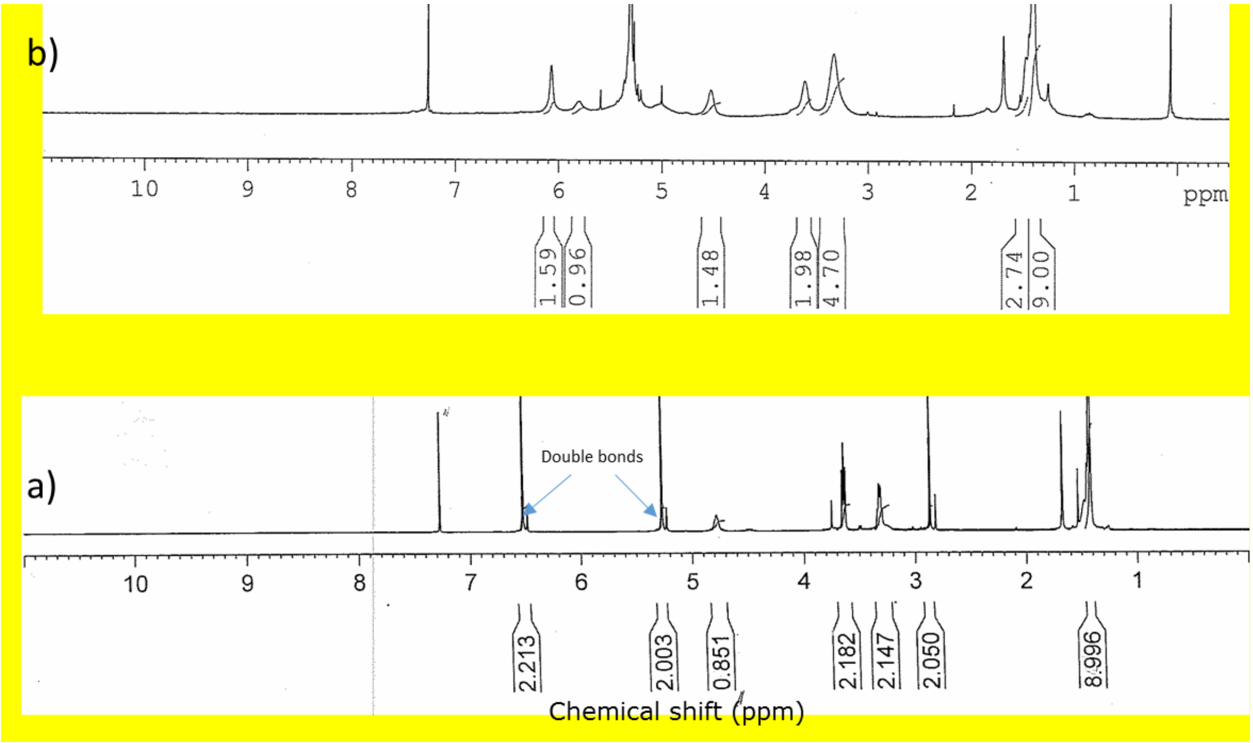

Figure S2. ^{1}HNMR spectra monomer a (bottom) and homopolymer spectra of Poly1a. NMR spectra were recorded on a Varian (600 MHz) NMR Spectrometer in deuterated chloroform.

Gel permeation chromatography. GPC analysis was done on polymer samples using THF on a 3µm SEC 200 (300 x 7.8 mm) column and an ELS detector. A flow rate of 1 ml/minute and a pressure of 60 PSI were used. Five poly(styrene) (Sigma-Aldrich) were used for calibration.

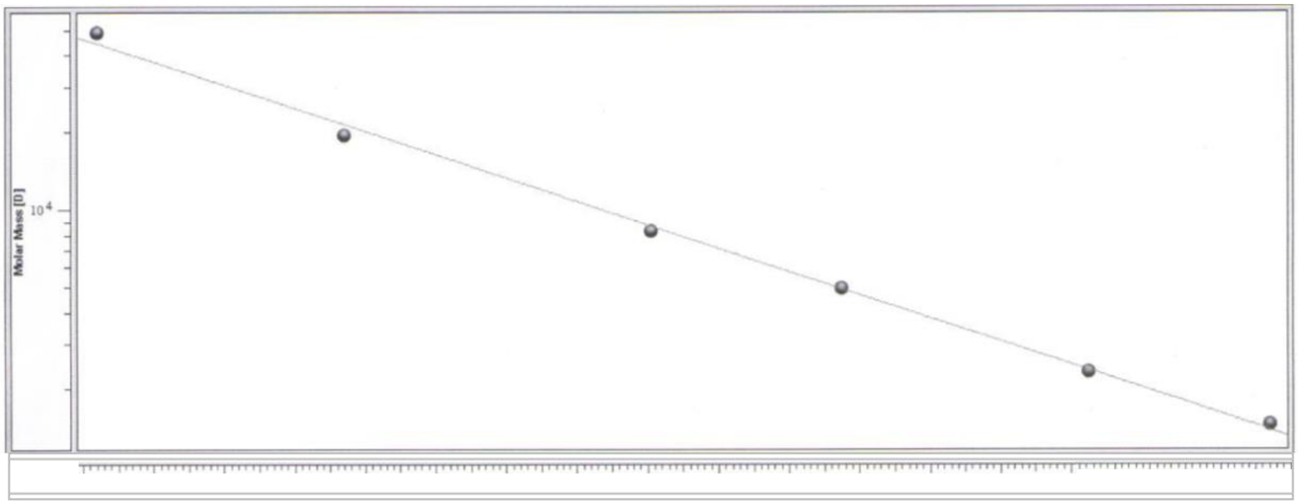

Elution Volume (ml)	Molar mass (D)
6.16552	500000
7.0345	198880
7.576	101000
8.2747	89620
8.7913	50000
9.5236	15000

Figure S3. Graph of retention time versus estimated molecular weight. Red squares refer to commercially available poly(styrne)..

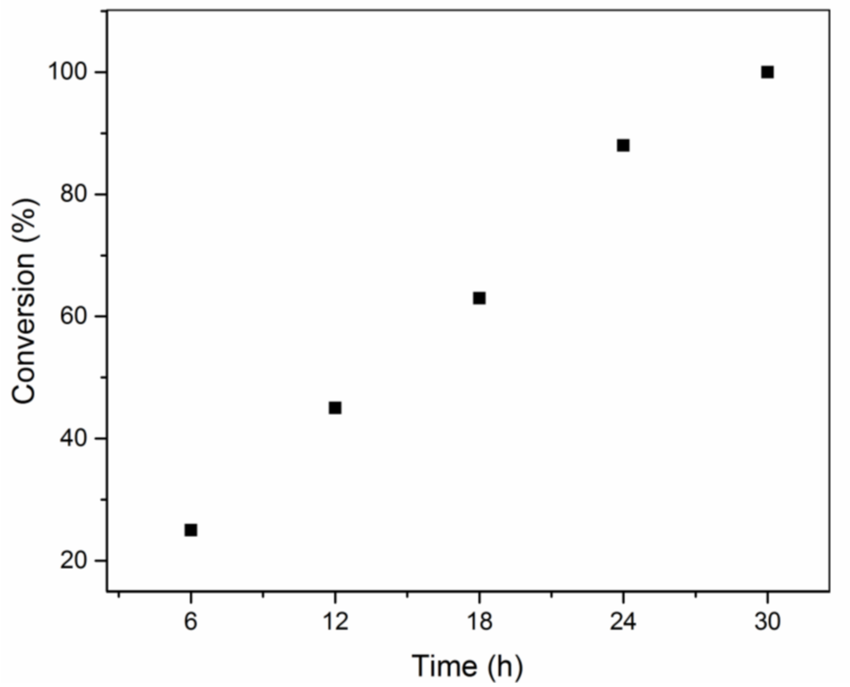

Figure S4. Graph of RAFT polymerization conversion table of macromonomer of Poly 2b and Poly2c.

AFM technique. AFM measurements was characterized on substrates with a JPK AFM Nanowizard. Commercial tips (Bruker) with a resonance frequency of 50 kHz and a spring constant of 0.10 N/m, were used to scan in contact mode all over the samples.
